# History of the Cholera Observed in Constantinople during the Winter of 1847—8

**Published:** 1848-05

**Authors:** 

**Affiliations:** Professor Agrégé of the Faculty of Paris


*History of the Cholera observed at Constantinople during the winter of* 1847-8. By M. Monneret, Professor Agrégé of the Faculty of Paris.—When I left France in November, 1847, the epidemic had been arrested at Trebizond, situated on the oriental shores of the Black Sea; this city had suffered cruelly. The disease had not extended beyond Kerezoun, also on the Black Sea, a little to the westward of Trebizond. On October 24th, it appeared at Constantinople without showing itself in the cities intermediate between that capital and Kerezoun.

When I arrived in Constantinople, at the beginning of December, the cholera morbus had attained a considerable degree of intensity. The soldiers were first affected, and at a later period the sailors of the Turkish fleet, which had recently entered the harbor. On the 18th forty-nine cases occurred on board one ship; the patients were removed to a temporary hospital belonging to the naval service. Up to January 5th, 248 patients were admitted into this hospital, and 73 died. Towards the same period 172 soldiers had been received into the military hospitals, and of these 83 cases proved fatal. It would be difficult to state accurately the precise proportion of the other inhabitants who were affected, but I am inclined to think that, in stating that altogether 1500 cases occurred, I am not far from the truth. My observations have been made in the military and naval hospitals; they have led me to the recognition of essential differences between the cholera of Constantinople and the Parisian epidemic of 1832. I now place these researches before the academy.

The comparison of cases observed at Constantinople points out three distinct forms of the disease. The first is constituted by cholera complicating other maladies, the second is cholerine, and the third differs little from Indian cholera. The symptoms, progress, and duration of treatment of these forms require a separate description.

1. *Cholera as a Complication.*—It would seem that in the incipient stage of the epidemic the specific influence was so weak that it was incapable of producing cholera unless assisted by some other affection, acting as a predisposing cause. It was, therefore, in patients suffering already from other disease that I first observed the development of cholera morbus. Occasionally it showed itself during the progress of some acute malady, such as pneumonia, bronchitis, pleurisy, simple or ulcerous colitis, dysentery; or during some chronic affection, phthisis, diarrhœa, or disease of the heart. In such cases the following symptoms were present:—Vomiting and diarrhœa, generally of bilious or serous matter, and, in some few cases, of the special characteristic, fleecy evacuations. Vomiting was not constant, and in some cases even constipation was observed; the tongue was pale, moist, and cold; the abdomen soft, and generally tender on pressure, particularly at the epigastrium. The face was altered; the expression of the eye vacant; the intellects dull, but present,

I carefully studied, in the patients affected with this form of cholera the three phenomena called algor, cyanosis, aud cramps. The face, hands, and feet, often the abdominal walls, were the seat of cyanosis and cold, which seldom invaded the entire body, and were usually less marked than in Asiatic cholera. I have seen them occasionally persist, during five or six days with some modifications induced by treatment. In others the blue colour was replaced by a sort of brick-tinged red hue. The pulse, weak and seldom insensible, resumed in some degree its strength when reaction appeared; this reaction was not readily established, and followed the progress of the primary disease which cholera had complicated. I have, for instance, in several patients affected with pneumonia, seen the cyanosis rapidly arrested under the influence of antiphlogistic treatment when the pulmonary inflammation subsided. In other cases I have noticed repeated diminutions and increases of cyanosis, according to the fluctuations of the principal malady, and, finally, too prolonged cyanosis was sometimes followed by an adynamic state, which gradually augmented, and brought on a fatal termination. It was thus that most patients died; others, in small numbers, were seized with tetanic convulsions, and expired during the phenomena of contraction, which seemed occasioned by congestion of the cerebro-spinal axis. The urinary secretion was suppressed. Cramps were uncommon in that form of cholera which we now refer to. The agitation of the patients was due to the intense abdominal sufferings.

Few patients affected with this cholera outlived the double malady which they laboured under; the mortality was, therefore, very great. It is doubtless very difficult to assign to each disease its share in the fatal issue; it may, however, with some probability, be admitted that the choleriform symptoms above described were in themselves insufficient to occasion death. Patients of robust constitution, and suffering from acute disease, recovered in larger proportions than the remainder.

*Second Form, of Cholerine.*—This form was much more frequent than Indian Cholera, and showed itself towards the end of December amongst the sailors. In proportion as the disease extended to a greater number of individuals, its effects seemed to grow weaker, and the attacks of algid cholera became more frequent. The greater number of sailors who at a later period suffered from Asiatic cholera, or from fatal cholerine, belonged to the Turkish squadron, which re-entered the harbour of Constantinople on the 1st of December, after a three months' cruise in the Archipelago. They were subject to diarrhœa, indigestion, caused by bad food, to nostalgia, and other morbid causes, which we will notice. Under these unfavourable conditions, cholerine, having existed for one or more weeks, was replaced by an attack of slight or fatal cholera. The symptoms of this cholerine, altogether similar to those observed at Paris, require no peculiar description.

*Third Form, Asiatic Cholera.*—Its symptoms had, in their intensity, duration, and association, some special characters deserving of attention. The cyanosis, often intense, and similar to that observed in the most violent cholera, was almost always partial, aad limited to the feet, face, hands, and forearms; it often ceased in twenty-four or thirty hours, without the patient being therefore out of danger. The skin of the cyanosed parts was viscid and wrinkled, as if macerated with water; the nails were often blue. The apparent refrigeration of the hands and feet afforded a contrast with the results of thermometric observation. In ten patients I examined the temperature at the axilla, and, if the thermometer was left a sufficint time, the mercury rose to 36° (96½,) and less frequently to 37° (98.) Under the influence of therapeutic agents capable of restoring heat the refrigeration speedily ceased; during several days, however, the calorification of the subject remained weak and uncertain until convalescence was properly established. I have assured myself that in many patients who lapsed into the adynamic state, although cyanosis never disappeared altogether, the skin remained hot and burning to the last.

Cramps were observed in about one-fourth of the cases: the tongue was moist and cold, but never blue. During the adynamic period it was red towards the apex, dry, viscid, and smooth; thirst was incessant, vomiting not frequent, and the rejected matter bilious, often fleecy and resembling rice-water. The abdomen often had the appearance special to Asiatic cholera; but, what, in my opinion, constitutes an important difference is, that in many subjects the belly was, on the contrary, voluminous, swollen, distended with gas, sonorous in the course of the large intestines, and tender on pressure in the epigastric and umbilical regions. These abdominal symptoms were present in so many subjects affected with violent cholera, that they might be considered as characteristic, and required a special mode of treatment.

In all cases in which I sought for the symptom, I met with intestinal "gargouillement" (enterophony.) The number of evacuations was not considerable, if compared with those of Indian cholera: in twenty-four hours the patients had seldom more than eight or ten motions. The matter was bilious or serious, sometimes fleecy and characteristic.

In bad cases the urine was almost invariably suppressed during several days; the respiration was frequent and laborious; some patients complained of considerable pain in the diaphragmatic insertions. During the period of re-action the pulse seldom exceeded 72; it rose to 108, and even 120, in the adynamic state.

After venesection the blood formed a black mass, which incarcerated the serum; the clot was soft, fragile, and presented no trace whatever of a fibrinous crust. In general, unless phlebotomy was performed during the first hours of the seizure, the blood flowed readily and even in stream from the venous orifice.

In mild cases, re-action was promptly established, and, if it was complete, convalescence soon set in; if, on the contrary, the cyanosis persisted, or diminished only in a slight degree, an adynamic condition was the result. This state, usually fatal, was marked by red patches on the face and hands, and difficulty of recovering heat; stupor or drowsiness, dulness of the eyes, convulsive closure of the lids, and dryness of the lips were also present. On the skin were observed no stains or typhoid eruption; epistaxis was not noticed; the spleen retained its natural size; and I have often at this period detected in both lungs sibilous or snoring rhonchi.

The extreme frequency of this adynamic state, which replaced the symptoms of cholera and even of simple cholerine, caused much anxiety to the physicians of the naval hospitals, who entertained fears of an early appearance of typhus in the narrow wards encumbered with a too large number of patients.

From the above description of the forms of the cholera at Constantinople, it follows that all the symptoms by which that malady is characterized were united in the greater proportion of the patients; these symptoms individually were, however, neither so intense nor so constant as in the cholera of 1832—the cyanosis, the refrigeration, and more especially, the cramps being less violent. . . .

Another important difference between the two epidemics consists in the predominance of abdominal symptoms, and the easy substitution of a generally fatal adynamic condition to cyanosis.

It would be hard to state what may have been the general mortality in Constantinople. One half of the soldiers, and rather less than one third of the sailors, affected were carried off by the disease.—*Ibid.*

